# Radiation doses from ^161^Tb and ^177^Lu in single tumour cells and micrometastases

**DOI:** 10.1186/s40658-020-00301-2

**Published:** 2020-05-19

**Authors:** Mario E. Alcocer-Ávila, Aymeric Ferreira, Michele A. Quinto, Clément Morgat, Elif Hindié, Christophe Champion

**Affiliations:** 1 Centre Lasers Intenses et Applications, Université de Bordeaux – CNRS – CEA, Talence, F-33400 France; 2 CERVO Brain Research Center, Department of Biochemistry, Microbiology and Bioinformatics, Université Laval, Quebec City, G1J 2G3 Quebec Canada; 3 Instituto de Física Rosario, CONICET – Universidad Nacional de Rosario, Rosario, S2000 EKF Argentina; 4 Service de Médecine Nucléaire, Hôpital Haut-Lévêque, CHU de Bordeaux, Pessac, 33604 France

**Keywords:** Monte Carlo simulation, Targeted radionuclide therapy, Terbium-161, Lutetium-177, Micrometastases

## Abstract

**Background:**

Targeted radionuclide therapy (TRT) is gaining importance. For TRT to be also used as adjuvant therapy or for treating minimal residual disease, there is a need to increase the radiation dose to small tumours. The aim of this in silico study was to compare the performances of ^161^Tb (a medium-energy *β*^−^ emitter with additional Auger and conversion electron emissions) and ^177^Lu for irradiating single tumour cells and micrometastases, with various distributions of the radionuclide.

**Methods:**

We used the Monte Carlo track-structure (MCTS) code CELLDOSE to compute the radiation doses delivered by ^161^Tb and ^177^Lu to single cells (14 *μ*m cell diameter with 10 *μ*m nucleus diameter) and to a tumour cluster consisting of a central cell surrounded by two layers of cells (18 neighbours). We focused the analysis on the absorbed dose to the nucleus of the single tumoral cell and to the nuclei of the cells in the cluster. For both radionuclides, the simulations were run assuming that 1 MeV was released per *μ*m^3^ (1436 MeV/cell). We considered various distributions of the radionuclides: either at the cell surface, intracytoplasmic or intranuclear.

**Results:**

For the single cell, the dose to the nucleus was substantially higher with ^161^Tb compared to ^177^Lu, regardless of the radionuclide distribution: 5.0 Gy vs. 1.9 Gy in the case of cell surface distribution; 8.3 Gy vs. 3.0 Gy for intracytoplasmic distribution; and 38.6 Gy vs. 10.7 Gy for intranuclear location. With the addition of the neighbouring cells, the radiation doses increased, but remained consistently higher for ^161^Tb compared to ^177^Lu. For example, the dose to the nucleus of the central cell of the cluster was 15.1 Gy for ^161^Tb and 7.2 Gy for ^177^Lu in the case of cell surface distribution of the radionuclide, 17.9 Gy for ^161^Tb and 8.3 Gy for ^177^Lu for intracytoplasmic distribution and 47.8 Gy for ^161^Tb and 15.7 Gy for ^177^Lu in the case of intranuclear location.

**Conclusion:**

^161^Tb should be a better candidate than ^177^Lu for irradiating single tumour cells and micrometastases, regardless of the radionuclide distribution.

## Background

Targeted radionuclide therapy (TRT) uses radiopharmaceuticals to target and irradiate tumour cells [1]. TRT was introduced several decades ago with the use of ^131^I for treating thyroid cancer. More recently, the potential for TRT of several other radionuclides, including *β*^−^ emitters as well as Auger electron emitters, has been explored [2, 3]. In particular, ^90^Y and ^177^Lu have been linked to biological vectors and found various therapeutic applications, including targeted treatment of non-Hodgkin lymphoma, peptide receptor TRT of neuroendocrine tumours and PSMA ligands TRT of metastatic prostate cancer [1, 4–6].

TRT faces two challenges: the heterogeneity found in large tumours and the energy escape from very small tumours. Heterogeneity can be addressed by using medium- or high-energy *β*^−^ emitters to increase the cross-dose to cold areas. However, these medium- or high-energy *β*^−^ emitters deliver most of the radiation dose outside of the targeted cells and therefore can fall short of the required dose to eradicate micrometastases and single tumour cells. Indeed, there is an optimal tumour size for “curability” associated to each radionuclide [7–10]. For instance, it is suggested that the *β*^−^ particles emitted by ^90^Y (mean energy = 933 keV) are more effective against large tumours (28–42 mm), while the *β*^−^ emissions of ^177^Lu (mean energy = 133 keV) would be more adapted for eradicating tumours of about 1.2–3 mm diameter [7]. The mean energy of ^177^Lu is, however, still too high when considering micrometastases or single tumour cells, which can be undertreated and be a source of relapse. For an electron energy released per unit of volume of 1 MeV per *μ*m^3^, a 2-mm sphere would receive 128 Gy, while a 200- *μ*m sphere would receive 42 Gy, and a 20- *μ*m sphere would receive only 6.6 Gy [10]. Theoretical dose calculations suggested that ^161^Tb may outperform ^177^Lu [10–12]. The superiority of ^161^Tb over ^177^Lu has been observed in cell survival studies, as well as in studies on mice bearing small tumour xenografts [13–15]. ^161^Tb has many intrinsic properties that make it very interesting for TRT [16]. In addition to its medium-energy *β*^−^ spectrum (mean energy = 154 keV), ^161^Tb emits a much higher number of very low-energy Auger electrons (AE) than ^177^Lu, as well as conversion electrons (CE) with low energy (mostly ≤ 40 keV). These low-energy electrons have high linear energy transfer and confer ^161^Tb an advantage over ^177^Lu up to about 30 *μ*m from the decay site [12]. Low-energy electrons emitted by ^161^Tb have been shown to increase the local dose in tumours without exacerbating renal damage [17]. ^177^Lu and ^161^Tb share chemical properties as radiolanthanides; thus, similar radiolabelling techniques can be used for both [13, 16]. Moreover, no-carrier-added ^161^Tb can be produced via the ^160^Gd(n, *γ*)^161^Gd →^161^Tb nuclear reaction in the quantity and quality needed for clinical applications [16, 18]. ^161^Tb emits a small percentage of photons that can be useful for post-therapy SPECT imaging, as is the case with ^177^Lu. Finally, ^161^Tb is compatible with the concept of theranostics, i.e. a diagnostic match may be found among other terbium radioisotopes allowing imaging before therapy, while ^177^Lu lacks a useful companion diagnostic radionuclide [19, 20].

In previous works [10, 12], we evaluated the absorbed doses from uniform distributions of ^161^Tb in water-density spheres of different sizes and compare it to ^177^Lu, ^67^Cu and ^47^Sc. Following energy normalisation, it was found that doses delivered by ^161^Tb per MeV released were similar to the doses delivered by the other radionuclides for spheres > 1 mm, but an advantage emerged for ^161^Tb for spheres < 1 mm, that progressively increased as sphere size decreased.

In the current work, we extend the comparison between ^161^Tb and ^177^Lu to take into account the subcellular distribution of the radionuclide. We assessed the radiation dose to the nucleus of a single cell for various specific subcellular distributions of the radionuclides. We also studied the absorbed doses to the nuclei of cells within a small cell cluster mimicking a micrometastasis.

## Methods

We computed the absorbed doses from simulations performed with the Monte Carlo track-structure (MCTS) code CELLDOSE, which has been described and validated in previous publications [21, 22].

The decay characteristics of ^177^Lu and ^161^Tb were taken from the ICRP Publication 107 [23] and are presented in Table 1. The whole *β*^−^ spectra were taken into account as well as all CE and AE emissions with probability greater than 0.0001. Photons were neglected.

**Table 1:** Decay characteristics of ^177^Lu and ^161^Tb

Radionuclide	^177^Lu	^161^Tb
Half-life (day)	6.647	6.906
Type of decay (%)	*β*^−^ (100%)	*β*^−^ (100%)
*β* particles mean energy (keV)	133.3	154.3
Daughter	^177^Hf (stable)	^161^Dy (stable)
CE (keV per decay)	13.52	39.28
CE energy range in keV (weighted average energy)^a^	6.2 – 206.3 (87)	3.3 – 98.3 (28)
AE (keV per decay)	1.13	8.94
AE energy range in keV (weighted average energy)^a^	0.01 – 61.7 (1)	0.018 – 50.9 (0.8)
Total electron energy per decay (keV)	147.9	202.5
*γ* for imaging: energy in keV (% abundance)	208 (11%); 113 (6.4%)	75 (10.2%)
Photons X and *γ* (total energy per decay in keV)	35.1	36.35

^a^The weighted average energy was computed as $\left (\sum _{i=1}^{n}{E_{i}*w_{i}}\right)/\sum _{i=1}^{n}{w_{i}}$, where *w*_*i*_ is the emission probability by nuclear transformation of an electron with energy *E*_*i*_

**Fig. 1:**
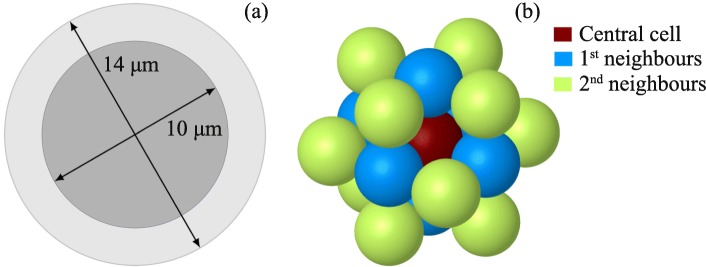
**a** Single cell of 14 *μ*m diameter with a nucleus of 10 *μ*m diameter and a total volume of 1436 *μ*m^3^. **b** Cell cluster as modelled in this work

The cell was modelled as a spherical volume of 14 *μ*m diameter, with a membrane of 10 nm thickness and a centred spherical nucleus of 10 *μ*m diameter (see Fig. 1a). All cell compartments were assumed to contain unit density water. The energy transferred by each electron to the medium was scored event-by-event until the electron’s energy fell below 7.4 eV (the electronic excitation threshold of the water molecule), in which case the remaining energy was considered as locally absorbed. Each simulation consisted of 1 million decays of the selected radionuclide (^177^Lu or ^161^Tb). We considered either of the following specific distributions of the radionuclide: only on the cell surface, only in the cytoplasm, only within the nucleus, and a uniform distribution in the whole cell. Because ^177^Lu and ^161^Tb do not have the same electron energy per decay (see Table 1), the absorbed doses were normalised considering that 1 MeV was released per *μ*m^3^ [10, 12]. This assumption means that for our cell of 1436 *μ*m^3^ volume, 1436 MeV were released from one of the regions of interest defined above. In our simulations, we assessed the absorbed dose to the nucleus, as the main critical target for radiation-induced cell death.

For the cluster, we considered cells arranged in a simple cubic structure model, as depicted in Fig. 1b. The cluster consisted of (i) a central cell, (ii) 6 cells forming the first neighbourhood in direct contact with the central cell and (iii) 12 cells forming the second neighbourhood. Given the symmetry of the system, the absorbed dose to a cell in a given neighbourhood is representative of the dose received by the other cells of that neighbourhood. Each cell of the cluster has the same dimensions as the isolated cell described above. All cells were assumed to be labelled in the same way, i.e. to contain a uniform distribution of the radionuclide in one of the specific regions of interest defined above (cell surface, or cytoplasm, or nucleus, or the whole cell). We assessed the radiation dose to the nucleus of the central cell, as well as to the nuclei of cells of the first and second neighbourhoods. In each case, we provide the self-dose, the cross-dose and the total dose. We did not simulate additional neighbourhoods: indeed, our previous results have shown that the superiority of ^161^Tb mainly resides in the emission of low-energy electrons [10, 12]. Let us note that all dose contributions to the cell nuclei of the cluster are due to electrons emitted from a distance equal to or less than the maximum diameter of our cluster (∼ 50 *μ*m).

## Results

### Single cell

We report in Table 2 the doses delivered by ^177^Lu or ^161^Tb. The absorbed dose to the nucleus of the single cell is lowest when the radionuclide is located on the cell surface, and highest when it is incorporated in the nucleus itself. However, regardless of the distribution of the radionuclide, the dose delivered by ^161^Tb is higher than the dose delivered by ^177^Lu. In order to facilitate the comparison between the two radionuclides, we also provide the enhancement factor, i.e. the absorbed dose ratio ^161^Tb/^177^Lu. As seen in Table 2 the enhancement factor is 2.6 in the case of cell surface location and up to 3.6 in case of intranuclear location.

**Table 2:** Absorbed dose^a^ (Gy) to the nucleus of a single cell for different distributions of ^177^Lu and ^161^Tb

	Cell surface	Intracytoplasmic	Whole cell	Intranuclear
^177^Lu	1.9	3.0	5.8	10.7
^161^Tb	5.0	8.3	19.5	38.6
Enhancement factor ^161^Tb/^177^Lu	2.6	2.8	3.4	3.6

^a^The absorbed doses computed with CELLDOSE correspond to a total electron energy release of 1436 MeV from one of the specific regions of interest of a cell of 14 *μ*m diameter and 10 *μ*m diameter nucleus

### Cell cluster

We report in Table 3 the absorbed dose to nucleus of the central cell within the cluster. As compared to the situation of the single cell (see Table 2), the addition of the 18 neighbouring cells increases the dose, regardless of the distribution of the radionuclide and more obviously so in case of cell surface distribution. Here again, it can be seen that the doses delivered by ^161^Tb are consistently higher than those delivered by ^177^Lu. More specifically, the enhancement factor ^161^Tb/^177^Lu is 2.1 in case of cell surface distribution and 3 in case of intranuclear location (see Table 3).

**Table 3:** Absorbed dose^a^ (Gy) to the nucleus of the central cell in a cluster for different distributions of ^177^Lu and ^161^Tb

	Cell surface	Intracytoplasmic	Whole cell	Intranuclear
^177^Lu	7.2 (26%)	8.3 (36%)	11.0 (53%)	15.7 (68%)
^161^Tb	15.1 (33%)	17.9 (46%)	29.1 (67%)	47.8 (81%)
Enhancement factor ^161^Tb/^177^Lu	2.1	2.2	2.6	3.0

^a^The target cell is surrounded by a first neighbourhood of 6 cells and a second neighbourhood of 12 cells. The absorbed dose was computed considering a total electron energy release of 1436 MeV from the specific regions of interest from every cell of the cluster. The relative contribution of the self-dose is shown in parentheses

We also estimated the dose to the nuclei of the cells of the first and second neighbourhoods. We give the absorbed dose as well as the percentage contribution of the self-dose (see Table 4). The relative contribution of self-dose increases as we move from the central cell to the first and second neighbourhoods. It also increases as we move from a cell surface distribution to an intranuclear distribution. For example, in the case of ^161^Tb, the self-dose contribution varies from 33% up to 90%. As Table 4 also shows, the dose from ^161^Tb is consistently higher than that of ^177^Lu, regardless of the distribution of the radionuclide and the position of the cell in the cluster.

**Table 4:** Absorbed dose (Gy) to the nucleus of any cell of the 1 ^st^ and 2 ^nd^ neighbourhoods, for different distributions of ^177^Lu and ^161^Tb

		Cell surface		Intracytoplasmic		Intranuclear	
		Neighbourhood		Neighbourhood		Neighbourhood	
	Cell position	1 st	2 nd	1 st	2 nd	1 st	2 nd
^177^Lu	Total dose in Gy	6	4.7	7	5.8	14.6	13.5
	Self-dose contribution	32%	40%	43%	52%	74%	80%
^161^Tb	Total dose in Gy	12.4	9.8	15.3	12.9	45.2	43.1
	Self-dose contribution	40%	51%	54%	65%	85%	90%

## Discussion

Cancer recurrence may occur months or years after surgery and is related to residual isolated tumour cells or micrometastases [24–26]. TRT can be very helpful as adjuvant therapy to eradicate such residual tumoral tissue. Adjuvant ^131^I therapy has been widely used in thyroid cancer patients. The concept of treating minimal residual disease with TRT has also been extended to other tumours [4, 27], for example as consolidation after chemotherapy in follicular non-Hodgkin lymphoma [4]. However, the radionuclides currently used for TRT in clinical practice have been designed for treating advanced disease, usually in patients with macrometastases and might not be optimal for adjuvant therapy. Indeed, with conventional radionuclides such as ^90^Y and ^177^Lu, most of the released energy would escape from single tumour cells or micrometastases, leading to reduced efficacy and increased toxicity. Other radionuclides may be more appropriate [7, 10, 12]. Among these radionuclides are alpha emitters and AE emitters. On the other hand, ^161^Tb can be of particular interest as it combines a medium-energy *β*^−^ spectrum similar to ^177^Lu with multiple emissions of AE and low-energy CE (see Table 1). These characteristics should allow using ^161^Tb in conventional situations of advanced disease, but also for adjuvant therapy. ^161^Tb has gained attention as an interesting alternative to ^177^Lu. The increased therapeutic efficacy of ^161^Tb over ^177^Lu has been demonstrated in both in vitro and in vivo studies [13–15], and clinical trials are currently being planned [15, 20].

**Fig. 2:**
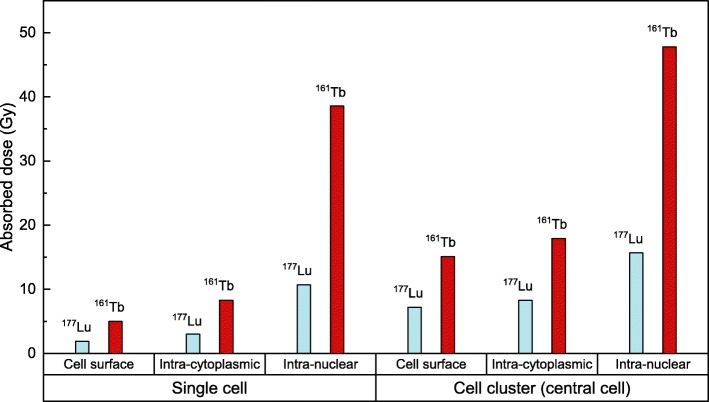
A comparison of absorbed doses delivered by ^177^Lu and ^161^Tb to the nucleus of a single cell and to the nucleus of the central cell in a cluster for different distributions of the radionuclide

In the present work, we used the MCTS code CELLDOSE to compute the radiation dose to the nucleus of isolated tumour cells and cells in a tumour cluster resulting from a specific distribution of the radionuclides in cell compartments. In each simulation, we considered 1436 MeV released (1 MeV per *μ*m^3^, see Fig. 1). Our study shows that the radiation dose delivered to the nucleus of a single tumour cell, and to the nucleus of any cell in a small cluster, is always higher with ^161^Tb than for ^177^Lu, regardless of the distribution of the radionuclide. Furthermore, for both radionuclides, the absorbed dose to the cell nucleus increases progressively as we move from a cell surface, to an intracytoplasmic and to an intranuclear distribution of the radionuclide. The radiation doses and enhancement factors for ^161^Tb and ^177^Lu are shown in Table 2 for the single cell and in Tables 3 and 4 for the tumour cluster. Figure 2 summarizes these results. These findings support the view that ^161^Tb may be a better choice than ^177^Lu for irradiating single tumour cells and micrometastases. On the other hand, it should be noted that even large tumours may benefit from a treatment with ^161^Tb. Indeed, large tumours are known to suffer from significant heterogeneity (necrosis, fibrosis, stromal tissue) that reduces the efficacy of cross-doses. In this context, ^161^Tb may provide a local boost to labelled tumoral cells because of the significant self-dose component offered by low-energy electrons.

Table 4 gives information on the percentage contribution of the self-dose in the cluster.

As it can be deduced from Fig. 2 and Tables 2 and 3, the incorporation of ^161^Tb into the cell nucleus would be of great interest to fully take advantage of its superiority over ^177^Lu. The transport of a radionuclide into the cell nucleus is a complex task, since the radiopharmaceutical must be designed to overcome the biological barriers imposed by both the cell membrane and the nuclear envelope [28]. Many teams are working to enhance nuclear targeting of radiopharmaceuticals, either for molecular imaging of intracellular proteins or TRT with AE emitters [28]. Specific dosimetry to DNA that might result from DNA-targeting molecules labelled with ^161^Tb has not been assessed in the present study.

Additional proof on energy deposit in vivo, with comparison between ^161^Tb and ^177^Lu, would require experimental microdosimetry data at cellular and subcellular level, using techniques such as beta imagers or micro-autoradiography [29–31]. On the other hand, the size of cell and nucleus influences simulations’ outcome and absorbed doses in cell compartments. In the present simulation, the cell and nucleus diameters were of 14 and 10 *μ*m, respectively, which results in the nucleus to occupy ∼ 36% of the total cell volume. This value is higher than in normal cells, where the average diameter of the nucleus is approximately 6 *μ*m and the nucleus occupies about 10% of the total cell volume [32]. Morphologically, the cancerous cell is characterized by a large nucleus, while the cytoplasm is scarce. Many cancers are diagnosed and staged based on graded increases in nuclear size [33]. The ratio of nucleus volume to cell volume we chose for our simulation is in line with other works. For example, in the work by Goddu et al. [34], the spherical cells have a diameter of 10 *μ*m and contain a concentric spherical nucleus of 8 *μ*m diameter, which results in a V _nucleus_/V _cell_ of ∼ 50%. In the recent study by Tamborino et al. [35] with experiments performed on human osteosarcoma cells, the V _nucleus_/V _cell_ was estimated as ∼ 30% based on 4Pi confocal microscopy.

Finally, it is important to stress that changing a radiometal can lead to substantial and sometimes unpredictable modifications in the affinity of a ligand to its receptor [36–38]. As lanthanides, terbium and lutetium share very similar chemistry. Chelators such as DOTA are adequate for both radionuclides and affinity of some labelled molecules looked similar [13–15], but this needs to be further confirmed with additional radiopharmaceuticals.

## Conclusion

^161^Tb associates the traditional advantages of a medium-energy *β*^−^ emission spectrum with the additional benefit of a high localised dose provided by conversion and Auger electrons. This allows a higher dose to the targeted cells and their immediate neighbours. ^161^Tb would always deliver higher absorbed doses than ^177^Lu in single tumour cells and micrometastases regardless of the cellular distribution of the radionuclide.

## Data Availability

The datasets used and/or analysed during the current study are available from the corresponding author on reasonable request.

## Abbreviations

AE: Auger electrons
CE: Conversion electrons
DOTA: Dodecane tetraacetic acid
MCTS: Monte Carlo track-structure
PSMA: Prostate-specific membrane antigen
SPECT: Single-photon emission computed tomography
TRT: Targeted radionuclide therapy.
